# The continuous downgrading of malnutrition in the German DRG system: possible effects on the treatment of patients at risk for malnutrition

**DOI:** 10.17179/excli2019-1256

**Published:** 2019-06-12

**Authors:** Laura E. Stollhof, Jessica M. Braun, Christoph Ihle, Anna J. Schreiner, Johannes Kufeldt, Michael Adolph, Elke Wintermeyer, Ulrich Stöckle, Andreas Nüssler

**Affiliations:** 1 Department of Traumatology, BG Unfallklinik Tübingen, Siegfried Weller Institute for Trauma Research, Eberhard Karls Universität Tübingen

**Keywords:** malnutrition, G-DRG, activity reimbursement, NRS

## Abstract

It has been internationally recognized that malnutrition is an independent risk factor for patients' clinical outcome. A new mandatory fixed price payment system based on diagnosis-related groups (G-DRG) went into effect in 2004. The aim of our study was to demonstrate the importance of carefully coding the secondary diagnosis of "malnutrition" in the G-DRG system and to highlight how the economic relevance of malnutrition in the G-DRG system has changed from 2014 to 2016. 1372 inpatients at the Berufsgenossenschaftliche Unfallklinik (Trauma Center) in Tübingen were screened for the risk of malnutrition using Nutritional Risk Screening (NRS-2002). Patient data were compared with the NRS values collected during the study and a case simulation was carried out separately for each year. We used the codes E44.0 for NRS = 3 and E43.0 for NRS > 3. The ICD codes were entered as an additional secondary diagnosis in the internal hospital accounting system DIACOS to determine possible changes in the effective weight. In 2014 the highest additional revenue by far was calculated by coding malnutrition. For the 638 patients enrolled in the study in 2014, we were able to calculate an average additional revenue per patient coded with malnourishment of €107. In 2016, we were unable to calculate any additional revenue for the 149 patients enrolled. Although it is well known that malnutrition is an independent risk factor for poor patient outcomes, nationwide screening for a risk of malnutrition when patients are admitted to a hospital is still not required. For this reason, malnutrition in German hospitals continues to be insufficiently documented**. **Due to the continuous downgrading of diagnosis-related severity (CCL) of malnutrition in the G-DRG system in trauma surgery patients, it is no longer possible to refinance the costs incurred by malnourished patients through the conscientious coding of malnutrition. We assume that the indirect positive effects of nutritional interventions will have to be taken into account more in the costing calculations and possibly lead to indirect cost compensation.

## Introduction

Malnutrition has been proven by various studies and internationally recognized to be an independent risk factor for patients' clinical outcome (Imoberdorf et al., 2010[14]; Barker et al., 2011[3]; Ihle et al., 2017[13]). Malnourished patients have a higher postoperative complication rate featured by wound healing disorders and nosocomial infections (Schreiner et al., 2018[27]). Consequently, there is a correlation between malnutrition and prolonged recovery time and an increased risk of hospital readmission could be observed as well. Depending on the medical specialty, the prevalence of malnutrition ranges from 16 to 55 %, with the highest prevalence being reported in oncology, internal medicine and geriatrics (Imoberdorf et al., 2010[14]; Lim et al., 2014[20]; Cruz et al., 2017[5]; van Puffelen et al., 2018[31]). Meanwhile, the role of malnutrition and even the risk of malnutrition is increasing in trauma surgery as well. Considering the demographic change and the increasingly older patient population in all medical disciplines, no improvement in the high prevalence of malnourished patients in German hospitals is to be expected (Statistisches Bundesamt, 2016[28]; Ihle et al., 2017[12]).

Since 2004 a new mandatory fixed price payment system based on diagnosis-related groups (G-DRG) is implemented in German hospitals (Hauser et al., 2004[11]; Bauer and Ostermann, 2012[4]). The goal was to create more transparency in the hospital accounting system, improve the effectiveness of work processes, and minimize costs. In the G-DRG system, an existing concomitant disease (e.g.; a patient with malnutrition) can only be billed as a secondary diagnosis or procedure if patient management is to include therapeutic or diagnostic measures or there is an increased need for support, nursing care and/or monitoring (Vogl, 2012[32]). Since there is a general agreement in the literature and professional groups, such as ESPEN/DGEM, that systematic screenings combined with a suitable nutritional approach can improve the outcome of malnourished patients after surgery (Klek et al., 2017[15]), the importance of malnutrition in the G-DRG system has been frequently discussed in recent years. In the present study, we evaluated the prevalence of malnutrition in a trauma surgery patient cohort with the aim to demonstrate the importance of accurate coding of the secondary diagnosis of "malnutrition" in the G-DRG system and to highlight the changes of the economic relevance of malnutrition in the G-DRG system from 2014 to 2016. 

## Materials and Methods

Between 01/2014 and 07/2016, 1372 inpatients at the Berufsgenossenschaftliche (BG) Unfallklinik (Trauma Center) in Tübingen were screened for the risk of malnutrition using Nutritional Risk Screening (NRS-2002), an international interview-based questionnaire for detection of malnutrition or a risk of malnutrition (Kondrup et al., 2003[16]). The data collection for this study was approved by the Ethical Committee of the University of Tübingen (Ethic Vote No. 429/2014BO2). Additional information about the study has already been published elsewhere (Ihle et al., 2017[12]; Ihle et al., 2017[13]; Lambert et al., 2017[18]). In this article we present a retrospective data analysis of the collected data. As part of the study design, the patient data were compared with the NRS values collected during the study and case simulations were carried out for each year separately. We used the codes E44.0 [moderate protein-energy malnutrition] for NRS = 3 and E43.0 [unspecified severe protein-energy malnutrition] for NRS > 3. The ICD codes were entered as additional secondary diagnosis in the internal hospital accounting system DIACOS to determine possible changes in the effective weight. The revenue changes were determined using the base case value valid for the respective year. We also simulated the billing as suggested by the working group of Reinbold et al. (2013[26]), by using the E44.0 code for NRS = 3 and the codes E43 for NRS ≥ 4 and E44.1 [mild protein-energy malnutrition] for NRS = 2. The applied coding guidelines are shown in Table 1(Tab. 1).

The collected data were analyzed using the jmp® 13.0.0 statistics program (SAS Institute Incorporation, Cary, NC/USA). 

## Results

### Clinical features of the cohort

Between 07/2014 and 07/2016, 1372 inpatients at the BG Unfallklinik in Tübingen were screened for the risk of malnutrition using the NRS-2002 screening tool (Ihle et al., 2017[12]). The clinical features of our study population are summarized in Table 2(Tab. 2). In terms of gender and age there was no significant difference between our study cohort and the hospital's general patient data. Although our study cohort included more patients who were in inpatient treatment in septic surgery or arthroplasty compared to the overall patient cohort, the difference was so insignificant, that our results could be extrapolated to the total patient population.

### NRS score

The NRS score was used to assess the nutritional status. In our study cohort, 19 % of the patients showed a moderate to high risk of malnutrition (NRS ≥ 3) and were therefore classified as malnourished. According to the criteria of Reinbold et al. (2013[26]), the percentage of patients classified as malnourished increased to 51 %. Both calculations are shown in Table 3(Tab. 3) (Reference in Table 3: Reinbold et al., 2013[26]).

### Factors influencing nutritional status

**Figure 1:**
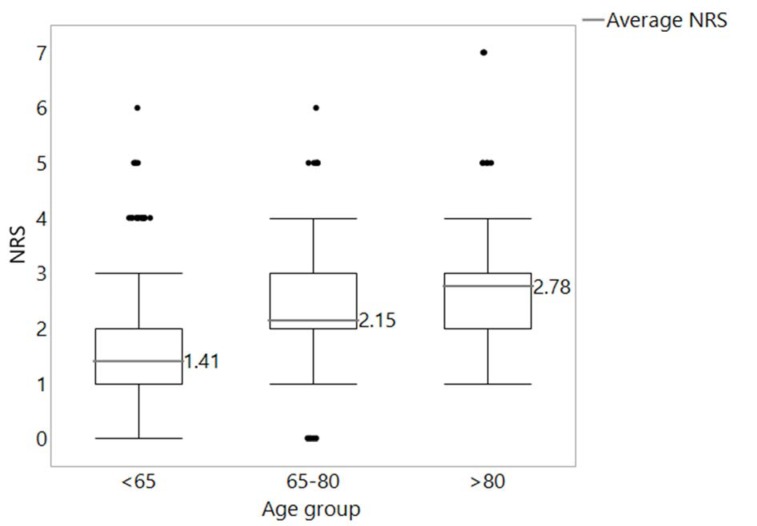
Distribution of NRS values in the age groups <65 years (n = 817), 65-80 years (n = 466) and > 80 years (n = 89). In black, the cut-off line for malnutrition according to Reinbold et al. (2013) (NRS ≥ 2)

Comparison of the distribution of malnutrition in different age groups indicated the correlation of increasing risk of malnutrition with a higher age. The results are shown in Figure 1(Fig. 1) (Reference in Figure 1: Reinbold et al., 2013[26]).

**Figure 2:**
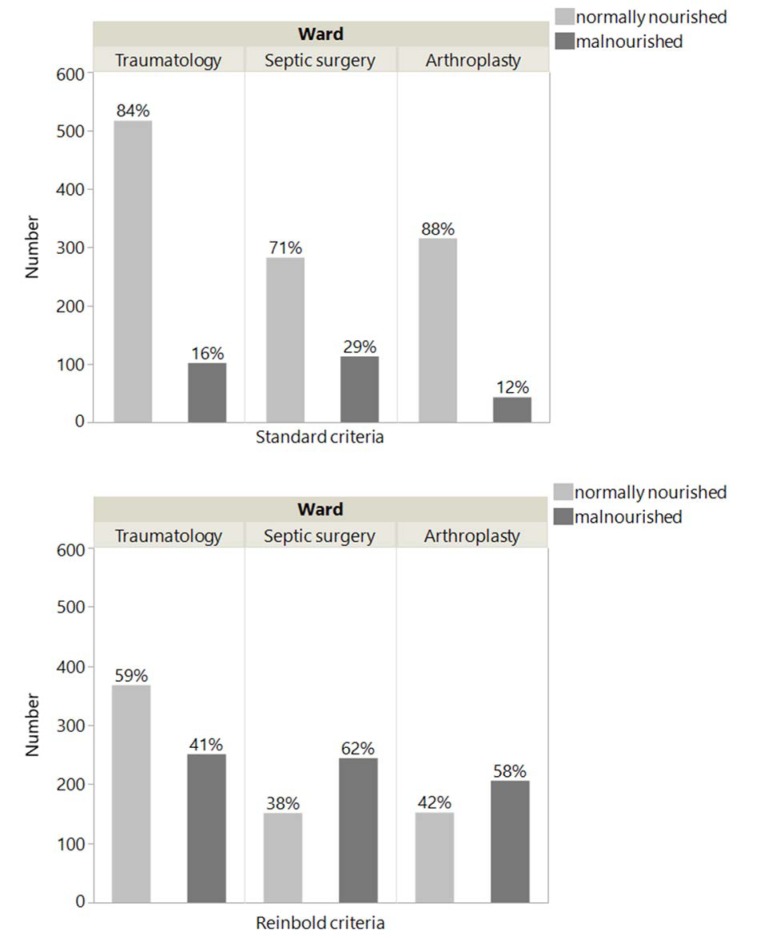
Proportion of malnourished patients with NRS ≥ 3 and NRS ≥ 2 at the traumatology, septic surgery and arthroplasty wards

If we calculate the percentage of malnourished patients of our study cohort based on inpatient treatment department (*e.g.*; trauma, septic surgery or arthroplasty), the following tendency could be observed: in septic surgery, 29 % of the patients are malnourished, while the percentage decreases to 16 % in traumatology and to 12 % in arthroplasty. The coding criteria of Reinbold et al. (2013[26]) changes numbers as follows: 62 % of patients in septic surgery, over 41 % malnourished patients in traumatology and 58 % of patients in arthroplasty (Figure 2(Fig. 2)).

### Changes in revenue due to the coding of the nutritional status

The coding of malnutrition as a secondary diagnosis leads to a change in the revenue. By coding malnutrition as a secondary diagnosis with E44.0 [moderate protein-energy malnutrition] for NRS = 3 and E43 [unspecified severe protein-energy malnutrition] for NRS > 3, a total additional revenue of € 96,298.39 was calculated from 2014 to 2016. The DRG grouper of the respective year was always used for the calculation. The calculated additional revenue after criteria of Reinbold et al. (2013[26]) amounts up to € 290,207.17 (Table 4(Tab. 4)).

Generally, the highest additional revenues were calculated for male patients. In our study, 65 % of the additional revenue was generated with male malnourished patients. The same tendency was also observed when calculating the average additional revenue. The average revenue generated by each male patient was € 7,768 and increased to € 7,849.72 through the additional coding of malnutrition (NRS ≥ 3) and to € 8,322.05 according to the criteria of Reinbold et al. (2013[26]). Again, the DRG groupers of the respective year were always used for the calculations and the results were summed up. The data is summarized in Table 5(Tab. 5), together with the data of female patients presented here for comparison.

**Figure 3:**
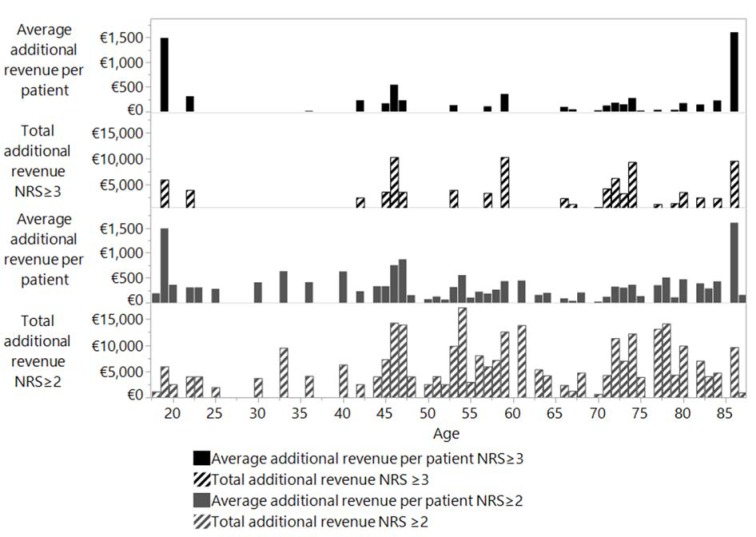
Revenue per person and total revenue in relation to patient age according to standard coding (NRS ≥ 3) and to Reinbold et al (2013) coding (NRS ≥ 2).

No correlation was calculated between average additional revenue from the coding of malnutrition and the age of the patients. However, considering the high number of patients over the age of 65 years in absolute terms, more additional revenue could have been calculated by encoding malnutrition. This correlation is shown in Figure 3(Fig. 3) (Reference in Figure 3: Reinbold et al., 2013[26]). 

Despite differences in patient numbers in the various departments (trauma, septic surgery or arthroplasty), the total revenue during our study period from 2014 to 2016 was equal in all departments. The highest proportion of 38.66 % in total revenue was achieved in the traumatology department with 619 treated patients. The septic surgery department accounted for 32.88 % of total revenue with 395 treated patients, whereas the arthroplasty department reached 28.46 % with the treatment of 358 patients. The DRG groupers of the respective accounting year were used for the calculations as usual and the results were summed up.

By conscientious coding of malnutrition according to the standard criteria, the percentage of revenue shifted to 79 % (equivalent to the additional revenue of € 76,073.94) for the septic surgery department, while only 21 % of total additional revenue have been earned in the traumatology department and no additional income have been earned in the arthroplasty department. The details are shown in Table 6(Tab. 6) in comparison with the coding criteria suggested by Reinbold et al. (2013[26]).

**Figure 4:**
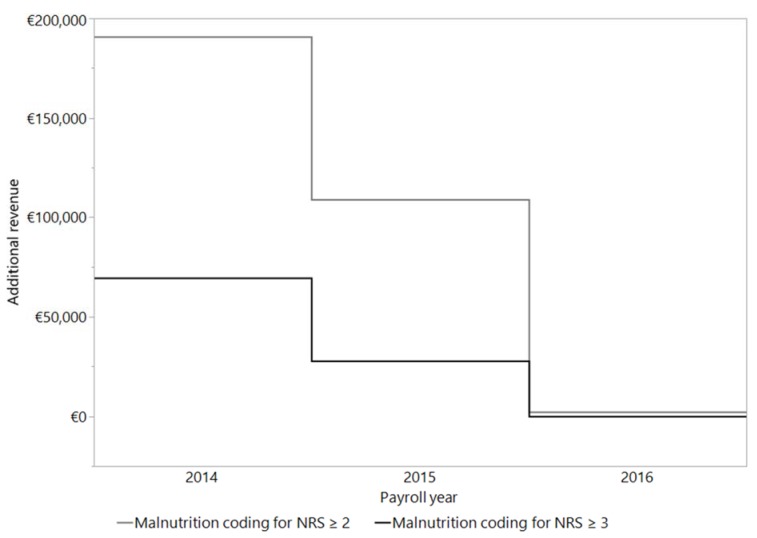
Calculation of the additional revenue per patient by coding the malnutrition (NRS ≥ 3 or NRS ≥ 2). The calculation was made with the DRG grouper of the respective years 2014 (n = 638), 2015 (n = 585) and 2016 (n = 149).

In 2014, compared to the following years of 2015 and 2016, the highest additional revenue by far was calculated by encoding malnutrition. This applies to both ways of coding the malnutrition - standard criteria as well as the criteria of Reinbold et al. (2013[26]). For the 638 patients enrolled in the study in 2014, we calculated an average additional revenue per patient coded with malnourishment of € 107 per standard coding and € 297 according to the coding criteria of Reinbold et al. (2013[26])*.* In 2015, 585 patients were included into the study, and the average additional revenue had fallen to € 47 (standard coding) or € 167 (coding criteria by Reinbold et al. (2013[26]) per patient. In 2016, no calculation of additional revenue could be made based on standard criteria for coding malnutrition for any of the 149 enrolled patients. Following the coding criteria of Reinbold et al. (2013[26])*, *an average additional revenue of € 15 per patient could have been calculated. The details of this comparison are presented in Figure 4(Fig. 4).

## Discussion

Although malnutrition is well known as an independent risk factor in patients' poor outcomes, screening for a risk of malnutrition at patients' admission to a hospital is not mandatory in Germany. For this reason, malnutrition in German hospitals continues to be insufficiently documented. This results in clinical disadvantages for the patients, who can face shortcomings in their treatment as well as in economic disadvantages for hospitals due to lost revenues for additional treatment (Barker et al., 2011[3]). The treatment costs caused by malnutrition in Germany are currently estimated at € 9 billion, and forecasts predict the continuous rise of these costs up to € 11 billion by 2020 (2013). 

Amaral et al. (2007[2]) found that malnourished patients cost a hospital about 20 % more than non-malnourished patients. Other research groups reported even higher additional costs of 31 to 50 % due to malnourished patients (Norman et al., 2011[22]; Gastalver-Martin et al., 2015[10]; Curtis et al., 2017[6]). Another point of discussion is the quality of the disease coding. Konturek et al. (2015[17]) screened 815 inpatients in 2015 and found that only 15 % of the cases of malnutrition were properly coded, resulting in a loss of approximately € 94,000 in their cohort. Ockenga et al. (2005[23]) reported even worse numbers in 2005. In their study a cohort of 541 gastroenterology patients, less than 1 % of the malnourished patients were conscientiously coded. In contrast, there are other studies and hospitals that routinely and thoroughly coded the malnutrition and could show that, with the right coding, the extra costs were at least partially offset by malnourished patients (Rasmussen et al., 2016[25]; Klek et al., 2017[15]). The AlHammoud and Reith research group at Constance Hospital showed that 419 screened patients out of 492 were malnourished and, if correctly coded would had been executed, an additional revenue of € 48 per patient could have been achieved (AlHammoud and Reith, 2013[1]). 

The financial burden of malnutrition was the focus of several other studies, and various published data demonstrated an annual deficit of between € 35,280 and € 184,032 for some relevant institutions (Löser, 2010[21]; Lim et al., 2014[20]; Konturek et al., 2015[17]; Leon-Sanz et al., 2015[19]; Thomas et al., 2016[30]). In contrast, other studies calculated additional revenue of more than € 70,000 from running a nutritional screening program, thereby refinancing the additional staff and material costs incurred by malnourished patients (Funk and Ayton, 1995[9]; Thomas et al., 2016[30]; Suarez-Llanos et al., 2017[29]). Still another study reported a reimbursement of 75 % of expenses caused by nutritional support, or 25 % loss of revenue in gastroenterology (Ockenga et al., 2005[23]). 

Most of the above studies were carried out and published in 1995 and 2010 to 2015. In our study, we observed a continuous downgrading of malnutrition in the G-DRG system from 2014 to 2016. It is important to note, that in all calculations revenue before deduction of the costs of personnel, materials and other hospital resources was considered. While additional proceeds of € 69,586 could be calculated for 2014, they dropped down to € 27,683 in 2015 and no additional revenues could be calculated 2016. If the coding guidelines of Reinbold et al. (2013[26]) are applied, the calculated additional revenue would increase to € 190,735 in 2014, followed by a drop in of additional revenues to € 108,939 in 2015 and € 2,304 in 2016 for the same number and disease state of patients. Our results clearly show that it has not been possible to reimburse the additional costs of malnourished patients since 2016, bearing in mind that we would like to monitor the patients' counseling, ONS and compliance. The lack of cost-effectiveness further reduces the incentive of the conscientious coding of malnutrition and can thus endanger patients. 

With regard to the clinical features, our study population was comparable to all patients at the site hospital and to data from the Federal Statistical Office's 2016 Hospital Report on gender, age, and reason for inpatient care (*e.g.*; trauma, septic surgery, or arthroplasty) (Statistisches Bundesamt, 2016[28]). We therefore assume that the above calculations could be extrapolated to the total population of inpatients. Consequently, in recent years an even higher profit could have been generated by the conscientious coding of malnutrition. 

Apart from other causes, such as depression and cancer, age is known to be a risk factor for the development of malnutrition (Eschbach et al., 2016[8][7]; Zhong et al., 2017[33]). Consistent with earlier findings, we observed a higher average age with women and a higher incidence of malnutrition with increasing age (Statistisches Bundesamt, 2016[28]; Ihle et al., 2017[12]; Lambert et al.*,* 2017[18]). The higher incidence of patients at risk for malnutrition among the elderly must be of growing interest in times of demographic change. One limitation of our study is that the prevalence of malnourished patients in our cohort may have been underestimated because of the inclusion of critically ill patients or exclusion of patients with dementia. Few studies have been conducted on dementia and malnutrition up to now. However, Eschbach et al. (2016[8]) showed an increased prevalence of malnutrition in patients with cognitive impairment. The largest percentage of malnourished patients was observed in septic surgery (29 %). This data confirmed previously published results from other studies that showed a strong negative association between malnutrition and wound (Barker et al., 2011[3]; Ihle et al.*,* 2017[13]; Suarez-Llanos et al., 2017[29]). We also calculated higher additional charges for men compared to women. This could be due to the higher percentage of men being treated in septic surgery. Based on our earlier published data and considering the here presented data along with all economic aspects, it should be possible to implement a nutritional screening and treatment at least for apparent high-risk groups (septic surgery and geriatric trauma) since these patients will definitely profitate from a treatment. 

Although the prevalence of malnutrition is being reported broadly in the literature, one of the main problems is still a lack of an internationally accepted criterion for the diagnosis of malnutrition and the use of various screening tools (Cruz et al., 2017[5]; Orlandoni et al., 2017[24]). As recently reported by Ihle et al. (2017[13]), both the MNA (Mini Nutritional Assessment) and the NRS appear to be suitable tools to detect a risk of malnutrition. Both screening tools are recommended by the professional associations ESPEN (European Society for Clinical Nutrition and Metabolism) and DGEM (German Society for Nutritional Medicine) and can be used in everyday clinical practice. Unfortunately, the current coding guidelines do not give any practical clues for the codification of a risk of malnutrition. There is no exact reference to translate the NRS into an ICD code. To be consistent with previous studies, we decided to do our calculations according to the coding suggestions of Reinbold et al. (2013[26]) as well as the standard coding criteria. By comparing them, we observed large differences between these two coding criteria in regard of the interpretation of NRS values (in other words the standard of NRS ≥ 3 (18.8 %) vs. NRS ≥ 2 (51.09 %)). A consensus on the coding guidelines would help to avoid this discrepancy. Due to the continuous downgrading of diagnosis-related severity (CCL) of malnutrition in trauma surgery patients in the G-DRG system, and without the conscientious coding of malnutrition it is no longer possible to generate additional revenue and to reimburse the costs incurred by malnourished patients. We assume that the indirect positive effects of nutritional interventions must be taken more into account in the cost calculations and possibly lead to indirect cost compensation due to less infections, shortening of LOS. Optimum patient care and improved patient outcomes must be the main stimuli for the introduction of a nationwide nutritional screening into standard hospital care. In addition, nutritional assessments can be counted as quality parameters. An indispensable prerequisite for this is a standardized method of documentation and coding.

## Acknowledgements

We would like to thank Peter Rapp for his support by the case simulation. In addition, we like to thank Mrs. Svetlana Gasimova for editing and proofreading of the final draft.

## Conflict of interest

The authors declare that they have no conflict of interest.

## Figures and Tables

**Table 1:**
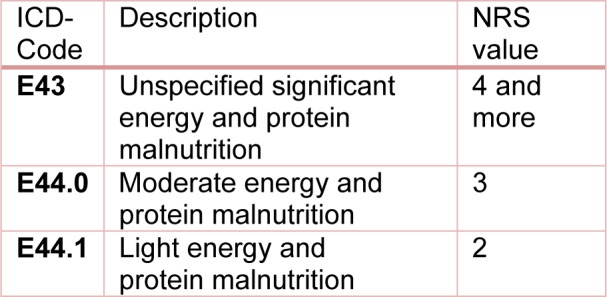
Coding guidelines according to Reinbold et al. (2013) for malnutrition

**Table 2:**
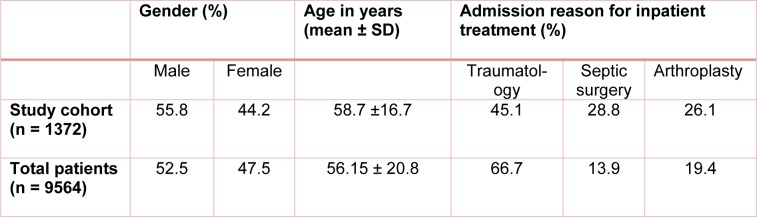
Clinical characteristics of the study cohort (n = 1372) and the total number of patients (n = 9564) hospitalized during the study period

**Table 3:**
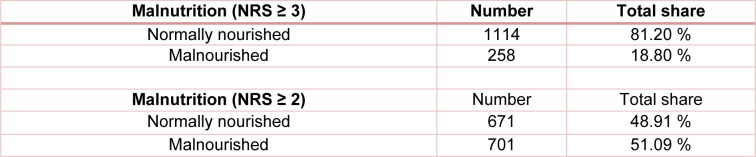
Malnourished patients according to standard criteria (NRS ≥ 3) and according to criteria of Reinbold et al. (2013) (NRS ≥ 2)

**Table 4:**
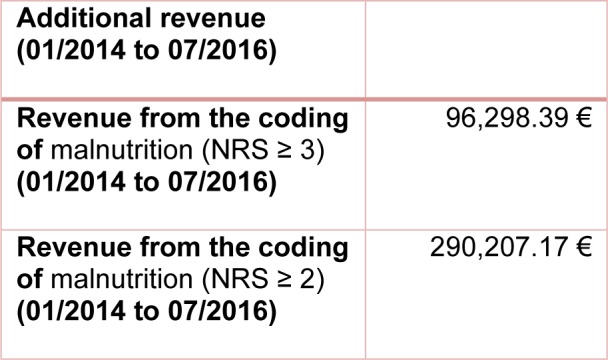
Additional revenue from codification of malnutrition from 01/2014 to 07/2016. The calculation was made with the DRG grouper of the respective year 2014 to 2016.

**Table 5:**
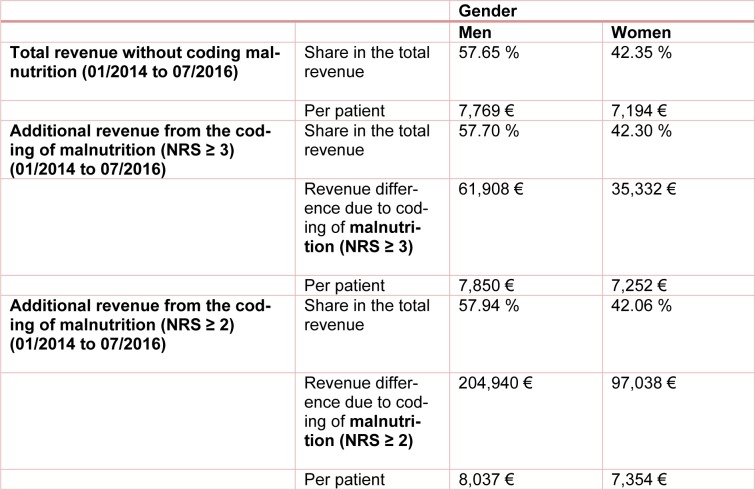
Distribution of total revenue and additional revenue per patient (male/female) with and without additional coding of the risk of malnutrition from 01/2014 to 07/2016. The calculation was made with the DRG grouper of the respective years 2014 to 2016.

**Table 6:**
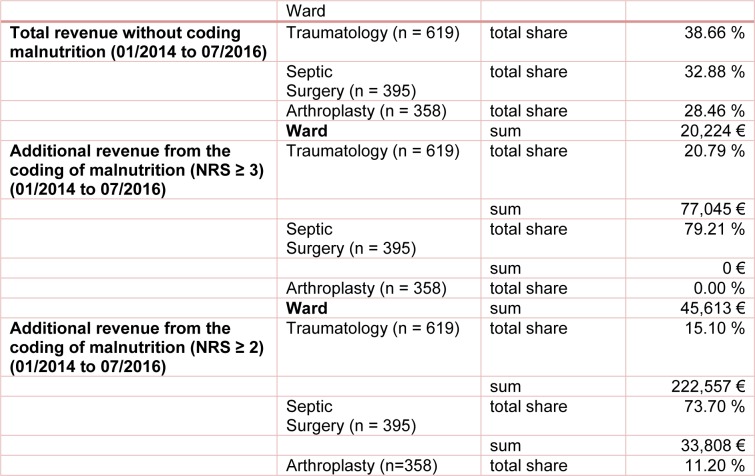
Distribution of total revenue with and without additional coding of malnutrition for NRS ≥ 3 and NRS ≥ 2 by ward. The calculation was made with the DRG grouper of the respective years 2014 to 2016.
